# Effect of oral clonidine premedication on perioperative haemodynamic response and postoperative analgesic requirement for patients undergoing laparoscopic cholecystectomy

**DOI:** 10.4103/0019-5049.76583

**Published:** 2011

**Authors:** Shivinder Singh, Kapil Arora

**Affiliations:** Department of Anaesthesiology & Critical Care, Armed Forces Medical College, Pune, Maharashtra, India; 1 Department of Anaesthesiolgy and Intensive Care, G. B. Pant Hospital, New Delhi, India

**Keywords:** Clonidine, laparoscopic, pain, pneumo-peritoneum, post-operative, stress response

## Abstract

Clonidine has anti-hypertensive properties and augments the effects of anaesthesia, hence we considered it to be an ideal agent to contain the stress response to pneumoperitoneum. We studied the clinical efficacy of oral clonidine premedication in patients undergoing laparoscopic cholecystectomies. Fifty patients scheduled for elective laparoscopic cholecystectomy under general anaesthesia were randomly allocated to receive premedication with either oral clonidine 150 μg (Group I, n = 25) or placebo (Group II, n = 25) 90 minutes prior to induction. The patients were managed with a standard general anaesthetic. The two groups were compared with respect to haemodynamic parameters, isoflurane concentration, pain and sedation scores, time to request of analgesic and cumulative analgesic requirements. Oral clonidine was found to be significantly better in terms of maintaining stable haemodynamics, having an isoflurane sparing effect and having a prolonged time interval to the first request of analgesia postoperatively compared to the control group. Administration of oral clonidine 150 μg as a pre-medicant in patients undergoing laparoscopic cholecystectomy results in improved perioperative haemodynamic stability and a reduction in the intra-operative anaesthetic and post-operative analgesic requirements.

## INTRODUCTION

Laparoscopic cholecystectomy was introduced by Phillipe Mouret in 1987.[1] Since then, it quickly became apparent that laparoscopy results in multiple benefits. In comparison with open procedures, laparoscopy is characterized by better maintenance of homeostasis.[2]

The hallmark of laparoscopy is creation of carbon dioxide (CO_2_) pneumoperitoneum and change in the patients position from Trendelenberg to reverse Trendelenberg. It also results in stress hormone responses (cortisol, epinephrine and nor-epinephrine) especially when CO_2_ pneumoperitoneum is used concomitantly.[3]

Clonidine is an α–2 adrenoreceptor agonist. It exerts central sympatholytic effect and has a half life of 9-12 h.[4] Premedication with clonidine blunts the stress response to surgical stimuli and the narcotic and anaesthetic doses are also reduced. In addition, clonidine increases cardiac baroreceptor reflex sensitivity to increase in systolic blood pressure, and thus stablises, blood pressure.[5]

These characteristics suggest that clonidine may be useful in the anaesthetic management of patients undergoing laparoscopic surgeries. Accordingly, this study was designed to evaluate the effects of oral clonidine premedication on haemodynamic response and modulation of post-operative pain in patients undergoing laparoscopic surgeries.

## METHODS

After approval from the Hospital research and ethical committee, a prospective, randomized, single-blind, comparative study was conducted on adult patients undergoing laparoscopic surgeries. Randomization was done using a computer generated random number table.

The study was conducted on 50 adult patients cases randomly divided into two groups of 25 each.

Group I patients were given clonidine 150 *μ*g po 90 min before induction.

Group II patients were given Vitamin C tablets 100 mg po 90min before induction.

### Inclusion criteria

ASA grades I and II, both males and females, adult patients aged 20 to 60 years, with scheduled Laproscopic cholecystectomy surgeries.

### Exclusion criteria

Patient not fulfilling eligibility criteria, lack of patient consent, drug dependence, history of bronchial asthma, patients allergic to clonidine, hypertensive and diabetic patients, severe coronary insufficiency, recent myocardial infection, concomitant use of monoamine oxidase inhibitors, tricyclic antidepressants or opioids.

During pre-anaesthetic assessment, a detailed history and examination of each patient was carried out to optimize them prior to surgery. On arrival in the operating room, all patients’ pulse oximetry, ECG and non-invasive blood pressure was recorded and a wide bore intravenous line established. Intra–operatively end tidal carbon dioxide (EtCO_2_), inspired and expired concentration of isoflurane was also measured.

The patients were pre-medicated with intravenous metoclopramide 0.1 mg/kg, ranitidine 1 mg/kg and fentanyl 2 *μ*g/kg. General anaesthesia was induced with thiopentone 5 mg/kg and vecuronium 0.1 mg/kg and maintained with isoflurane in 60% N_2_O/ 40%O_2_ mixture. Controlled mechanical ventilation was applied to maintain endtidal CO_2_ between 30-40 mmHg. The mean arterial blood pressure (MABP) was maintained at 20% above or below the pre-operative value by adjusting the concentration of isoflurane.

In case of severe haemodynamic fluctuations, medical intervention other than adjustment of isoflurane was applied. For bradycardia (heart rate lower than 60 bpm), atropine 0.6 mg i.v. was administered. Hypotension (MABP <60 mmHg) was managed with fluid challenges and/or i.v. mephentermine 6 mg bolus. Hypertension (MABP > 110 mmHg) was treated with inj. nitroglycerine 0.5-5*μ*g/kg/min i.v.

Haemodynamics and isoflurane concentration was recorded. Prior to induction, 1 min after endotracheal intubation, 5 min after endotracheal intubation, at skin incision, start of pneumoperitoneum, 15 and 30 min after institution of CO_2_ pneumoperitoneum and 15 min after release of pneumoperitoneum.

Pain intensity was assessed using a 10-cm visual analog scale (VAS). Zero denoting no pain and 10 denoting intolerable pain. Sedation was assessed using Ramsay sedation score from 1 to 6. Occurrence of any adverse events like nausea, vomiting, hypotension, hypertension, bradypnea and CO_2_ retention was recorded.

VAS, sedation score and adverse events were recorded at 30 min, 60 min, 90 min and 120 min postoperatively. Time of the first request of analgesic (TAR) signifying the period of time elapsed from the surgery to the time when the first dose of analgesia was given at patients request (VAS>4) was recorded. Rescue analgesia was given in the form of a combination of Inj. Diclofenac sodium 75 mg i.v. over 30 min 12 hourly and Inj. meperidine 0.5-l mg/kg i.m. 8 hourly. The total dose of post-operative analgesic was also recorded.

### Statistical analysis

The statistical significance for categorical variables was determined by chi-square test. Fisher exact test was used in case one or more expected cell count was less than 5. For continuous variables two sample t-test was applied. Non paramertric “Mann whitney” test was used for data that did not follow a normal distribution. Results were expressed as Mean ± SD (standard deviation). A *P* value < 0.05 was considered statistically significant.

Demographic data (age, weight, height), haemodynamic data (Heart rate, Mean arterial pressure [MAP]), isoflurane requirement were subjected to statistical analysis using two sample t-tests. For statistical analysis of the time to the first dose of post-operative analgesic, 24-h cumulative analgesic requirement, VAS and sedation score not normally distributed ‘Wilcoxon Mann Whitney’ test, which is a non-parametric test, was applied. Sex was analysed by chi-square test. Adverse effects were analysed by Fisher’s exact test. Taking α = 0.05, for detecting mean time of requirement of analgesic from the end of surgery (185.6 ± 34.13 minutes in group I vs. 100.40 ± 24.33 minutes in group II), taking sample size of 25 in each group, the power of study is approximately 74%.

## RESULTS

There were no differences between the clonidine and placebo groups regarding age, sex and weight [Table 1].

**Table 1:** Demographic data

	Clonidine (n = 25)	Placebo (n = 25)
Sex (M/F)	7(28%)/18 (72%)	4 (16%)/21 (84%)
Age (yr)	36.04 ±10.43	33.68 ± 7.83
Height (cm)	163.8 ± 3.42	162.08 ± 3.83
Weight (kg)	62.76 ± 4.10	62.08 ± 3.25

The basal heart rate was not comparable. There was a rise in pulse rate post-intubation the difference being statistically significant only at 5 min after intubation. Perioperatively the mean heart rate was lower in group I as compared to group II. Mean heart rate ranged from 79.28 ± 9.50 to 85.84 ± 10.12 in group I, whereas it ranged between 83.80 ± 12.76 to 100.04 ± 12.16 in group II. At skin incision, following start of pneumoperitoneum and 15 min after pneumoperitoneum changes in mean heart rate between the two groups was statistically significant (*P* value < 0.05) [Table 2].

**Table 2:** Heart rate

	Group I Mean ± SD	Group I Mean ± SD	*P* value
Baseline	83.8 ± 12.61	87.4 ± 13.22	0.33
1 min after intubation	85.84 ± 10.12	100.04 ± 12.16	0.33
5 min after intubation	81.86 ± 8.52	92.56 ± 8.84	0.001
Skin incision	79.36 ± 10.46	90.24 ± 9.55	0.001
Start of pneumoperitoneum	79.28 ± 13.69	89.20 ± 11.5	0.008
15 min	79.36 ± 10.74	88 ± 14.17	0.02
30 min	79.28 ± 9.50	83.80 ± 12.76	0.16
15 min after release	82.32 ± 9.49	87.04 ± 9.98	0.09

The basal MAP of the two groups was not comparable. The maximum rise in MAP was noted at 1 minute after intubation and at start of pneumoperitoneum in both groups. Perioperatively, the MAP was lower in group I compared to group II. MABP ranged from 88.77 ± 7.99 to 102.41 ± 10.35 in group I, whereas it ranged from 96.99 ± 6.37 to 114.8 ± 14.08 in group II. The difference in the MAP value between the two groups was significant at all time points except at 15 min after the release of pneumoperitoneum when it became not significant [Table 3].

**Table 3:** Mean arterial blood pressure

	Group I [Mean ± SD (mm Hg)]	Group II [Mean ± SD (mmHg)]	*P* value
Baseline	101.51 ± 8.16	100.32 ± 8.16	0.607
1 min after intubation	101.92 ± 10.45	114.8 ± 14.08	0.001
5 min after intubation	87.61 ± 8.36	96.99 ± 6.37	0.001
Skin incision	88.77 ± 7.99	97.39 ± 9.93	0.001
Start of pneumoperitoneum	102.41 ± 10.35	109.79 ± 11.27	0.002
15 min	100.75 ± 6.59	107.65 ± 8.37	0.002
30 min	97.17 ± 6.19	106.16 ± 7.76	0.001
15 min after release	99.02 ± 4.94	101.33 ± 4.78	0.10

**Figure 1:**
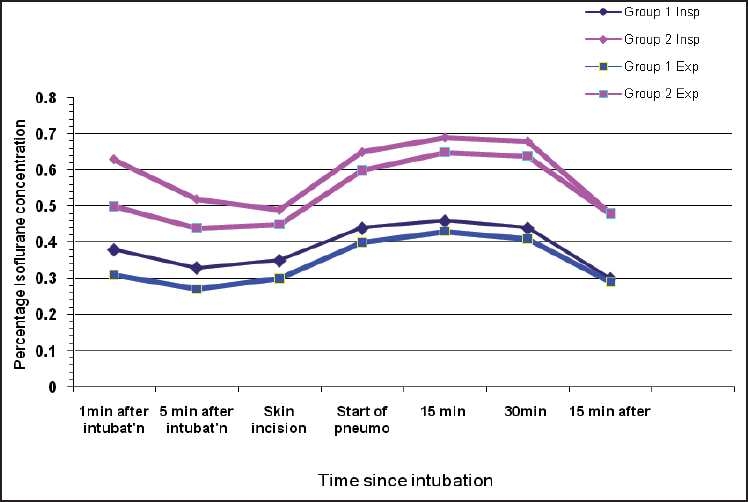
Comparison of isoflurane (percent concentration insp. and exp.) between group I and group II

The isoflurane concentration requirement at 1 and 5 min post-intubation was higher in group II than group I; the difference being statistically significant. During the rest of perioperative period isoflurane concentration required for maintaining acceptable haemodynamics was reduced in group I than group II and comparable (*P* < 0.05) [Figure 1].

There was no statistically significant difference between the two groups in VAS and sedation scores recorded at 30 min intervals till 2 h postoperatively.

TAR was significantly prolonged in the clonidine group in comparison with the placebo group (185 ± 34.4 vs 100.40 ± 24.3 min). There were more patients in the clonidine group that required analgesic 6-8 h postoperatively than in the control group (24% vs 8 %) who had already taken their first dose of analgesic.

Remarkably less patients were given a single dose of meperidine during the first 24 hours postoperatively in the clonidine group (72% v 32%, *P* < 0.05). On the contrary, more patients in the placebo group required 2 or more than 2 doses of meperidine (68% vs 28%, *P* < 0.05). Similarly, significantly less patients required only one dose of diclofenac sodium during the first 24 h postoperatively in clonidine group (92% vs 60 %, *P* < 0.05). Also, more patients in placebo group needed a second dose of diclofenac Sodium (40% vs 8%, *P* < 0.05) to alleviate post-operative pain.

Incidence of nausea and vomiting was less in the clonidine group compared to the control group (28% vs 52%) though not significant (*P* > 0.05). Bradycardia and hypotension were found only in the clonidine group and was also statistically insignificant [Table 4]. No other adverse effects were noticed.

**Table 4:** Adverse effects

	Group I (n = 25)	Group II (n= 25)	*P* value
Nausea	3 (12)	8 (32)	0.088
Vomiting	4 (16)	5 (20)	0.713
Bradycardia	4 (16)	0	0.054
Hypotension	3 (21)	0	0.117
Bradypnea	0	0	1

Figures in parentheses are in percentage

## DISCUSSION

During laparoscopic surgical procedures changes in the patient’s position and surgical stress, especially following pneumoperitoneum cause labile haemodynamics. The choice of anaesthetic technique for upper abdominal laparoscopic surgery is mostly limited to general anaesthesia with muscle paralysis, tracheal intubation and intermittent positive pressure ventilation.[6] This study was conducted in 50 adult patients belonging to ASA physical status I and II, to evaluate the effect of clonidine premedication on haemodynamic response and post-operative pain associated with laparoscopic cholecystectomy. Clonidine is rapidly and completely absorbed after oral administration and reaches peak plasma concentrations within 60-90 min. In our study, tablet clonidine was given 90 min before scheduled laparoscopy.

Hypertension and tachycardia were noticeable during the application of CO_2_ pneumoperitoneum in the placebo group. Patients premedicated with clonidine had more stable haemodynamics than those pre-treated with placebo. Clonidine premedication effectively blunted the cardiovascular response to surgical stress, especially pneumoperitoneum. Compared with the baseline measurements, there was significantly less increase in heart rate and MAP in the clonidine group compared to the placebo group.

Tablet clonidine in the dose of 150 mcg orally for premedication has been used by other authors who have documented maintenance of stable haemodynamics intraoperatively and during pneumoperitoneum.[8–10] Higher doses (5 *μ*g/kg) have also been used with a significant attenuation of responses to hypercapnia.[11] In our study, we have also used 150 *μ*g of clonidine orally.

A reduction of isoflurane requirement was observed in our patients who were premedicated with clonidine. They maintained desired haemodynamics at significantly lower concentrations of isoflurane. Our findings were in concordance with other studies in which there was decrease in MAC and inhalational agent requirement.[12,13] In another study, the pre-operative use of oral clonidine (3.5 *μ*g/kg) followed by IV infusion postoperatively was found to improve the haemodynamic profile associated with anaesthetic discontinuation, thus further proving its anaesthetic-sparing effect.[14]

In our study, TAR was prolonged in patients of clonidine in comparison with placebo group. Most of the patients in the clonidine group required no meperidine or only one dose during the post-operative 24-h period while more patients in the placebo group required 2 or more than 2 doses of meperidine. Similarly, more patients in the clonidine group required no diclofenac sodium or only one dose of diclofenac sodium during the post-operative 24-h period, while 2 or more than 2 doses were required in most of the placebo group patients.

**Figure 2:**
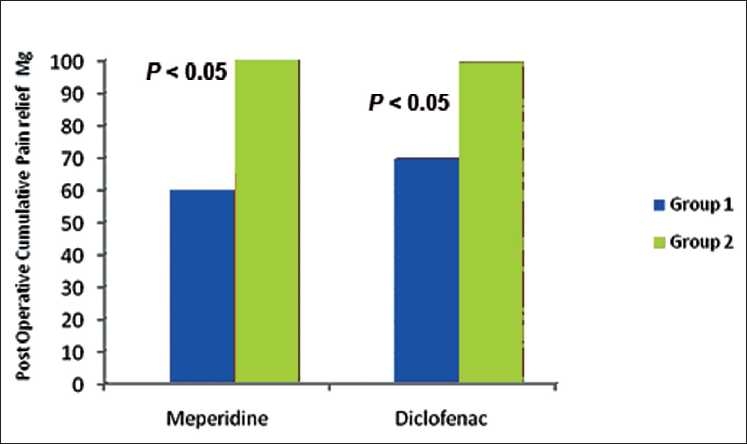
Post-operative 24 hour cumulative requirement

The cumulative analgesic requirement (in 24 h) of both meperidine and diclofenac was statistically significantly less for group I vs group II *P* < 0.05 [Figure 2](ranging from 60 ± 40.82 mg in group 1 vs 100 ± 50 mg in group II for meperidine and from 69 ± 36.99 to 99 ± 47.03 in group I and II, respectively for diclofenac), thus demonstrating a significant beneficial effect of clonidine. Other studies have similar findings. However, in a study comparing oral clonidine premedication with oral diazepam no statistical difference in the post-operative VAS scores for pain, number of analgesic requests and emesis was found.[17] Possibly since the comparison was not done with a placebo but with a hypnotic.

There was no significant difference in VAS at 2 h from the end of surgery between the two groups because as per protocol patients were given rescue analgesia the moment VAS was ≥4 and the patients were kept pain free. Moreover, the clonidine group patients were not significantly sedated compared to the control group, unlike other reports using a higher dose of clonidine where patients were found to be more sedated and the incidence of bradicardia was higher.[18]

To conclude, the administration of oral clonidine 150 *μ*g as a simple and cost effective form of premedication in patients undergoing laparoscopic cholecystectomy results in improved perioperative haemodynamic stability and reduction in anaesthetic requirements. In addition, it also reduces the post-operative analgesic requirements.
